# Induction chemoradiotherapy achieves long-term recurrence-free survival in locally advanced pulmonary lymphoepithelioma-like carcinoma: a case report and literature review

**DOI:** 10.3389/fimmu.2025.1605900

**Published:** 2025-07-31

**Authors:** Gengda Huang, Hong He, Yuting Fan, Qinqin Ren, Huixin Jiang, Li Luo, Li Guo, Haiwen Chen, Jiexia Zhang

**Affiliations:** ^1^ Department of Respiratory and Critical Care Medicine, State Key Laboratory of Respiratory Disease, National Clinical Research Center for Respiratory Disease, National Center for Respiratory Medicine, Guangzhou Institute of Respiratory Health, the First Affiliated Hospital of Guangzhou Medical University, Guangzhou, Guangdong, China; ^2^ School of Clinical Medicine, Guangzhou Medical University, Guangzhou, Guangdong, China; ^3^ Department of Pulmonary and Critical Care Medicine of Jiangbei Campus, The First Affiliated Hospital of Army Medical University (The 958th Hospital of Chinese People's Liberation Army), Chongqing, China

**Keywords:** non-small cell lung cancer, Epstein-Barr virus, lymphoepithelioma-like carcinoma, induction chemotherapy, radical radiotherapy

## Abstract

Pulmonary lymphoepithelioma-like carcinoma (pLELC) is a rare subtype of non-small cell lung cancer (NSCLC) that is closely associated with Epstein-Barr virus (EBV) infection. While radiotherapy after induction chemotherapy have shown marked efficacy in nasopharyngeal carcinoma (NPC), its effectiveness in pLELC remains uncertain. A female with stage IIIB pLELC was treated with 4 cycles of gemcitabine plus cisplatin, achieving partial response(PR), followed by radical radiotherapy. Patients achieved clinical complete response(CR) with recurrence-free for over five years. This case highlights the potential of induction chemotherapy as an effective treatment for locally advanced pLELC.

## Background

pLELC is a rare subtype of NSCLC, accounting for approximately 1% of newly diagnosed lung cancers (1). It was closely linked to Epstein–Barr virus (EBV) infection, meanwhile, its tumor microenvironment typically shows substantial lymphocytic infiltration, similar to other EBV-related malignancies, such as NPC (2, 3). In the most recent fifth edition of World Health Organization (WHO) classification of lung cancer, pLELC was categorized under squamous cancer. However, due to its rarity, no standard therapies or consensus of pLELC had been established, particularly for patients in unresectable locally advanced stage. The clinical management of pLELC typically follows treatment strategies for squamous cell lung cancer, with sequential chemoradiotherapy and definitive concurrent chemoradiotherapy being the common options for locally advanced squamous cell lung cancer. Due to the distinctive characteristics of pLELC, including its association with EBV infection, variable lymphocyte infiltration, and distinct genotype compared to squamous cell lung carcinoma, its sensitivity to radical concurrent chemoradiotherapy and sequential chemoradiotherapy may differ (4, 5). Notably, a phase III clinical trial has demonstrated that adding induction chemotherapy prior to radiotherapy significantly improves recurrence-free survival (RFS) and overall survival (OS) in patients with locally advanced nasopharyngeal carcinoma (NPC) (6). These findings established induction chemotherapy followed by radiotherapy as the standard treatment by clinical guidelines. However, despite the shared clinicopathological features between NPC and pLELC, the efficacy of induction chemotherapy combined with radiotherapy remains underexplored in pLELC. We present the first documented case of radiotherapy induction chemotherapy followed by radical radiotherapy for pLELC. This report aims to provide preliminary evidence and insights into the treatment of unresectable, locally advanced pLELC.

## Case presentation

**Figure 1:**
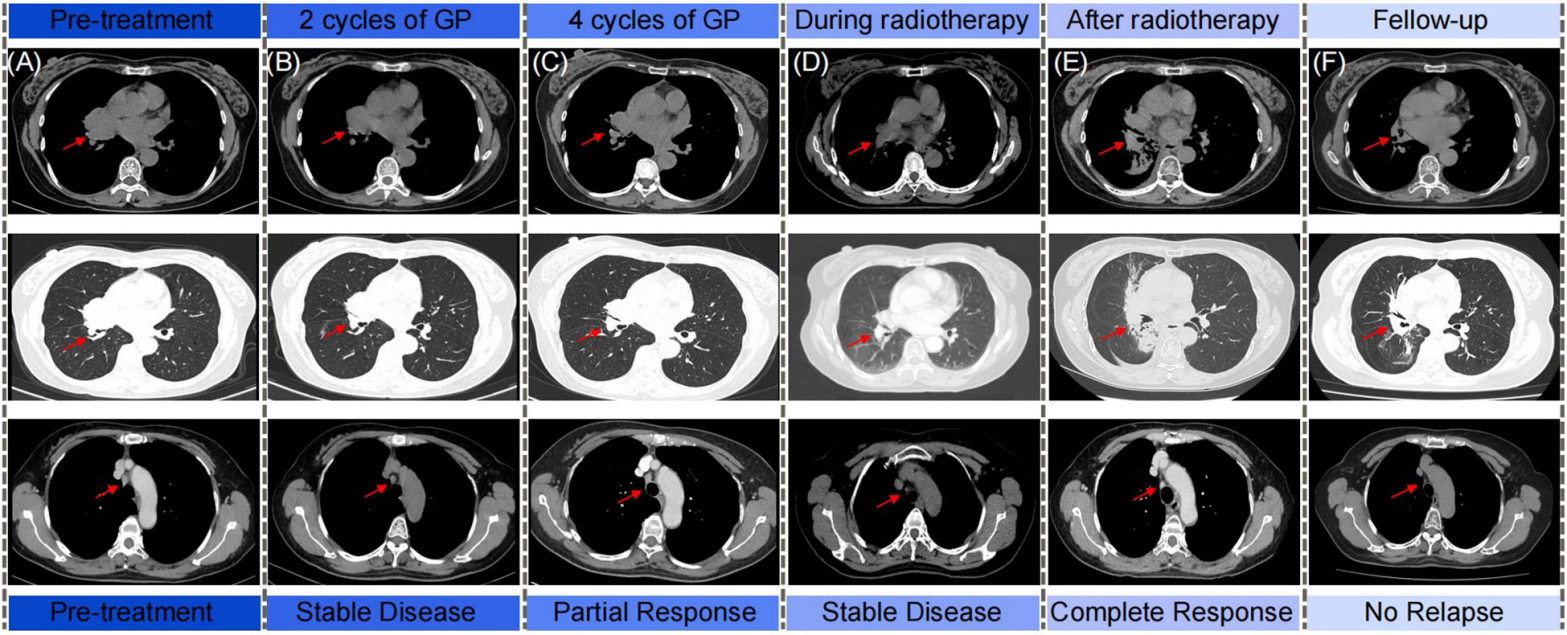
The schematics show the computed tomography scan from diagnosis, chemotherapy, radiotherapy to follow-up of patient. Rad arrows show the primary chest tumor and the regional lymph nodes. **(A)** Pre-treatment; **(B)** After 2 cycles of GP chemotherapy; **(C)** After 4 cycles of GP chemotherapy; **(D)** During radiotherapy; **(E)** After radiotherapy; **(F)** Follow-up.

**Figure 2:**
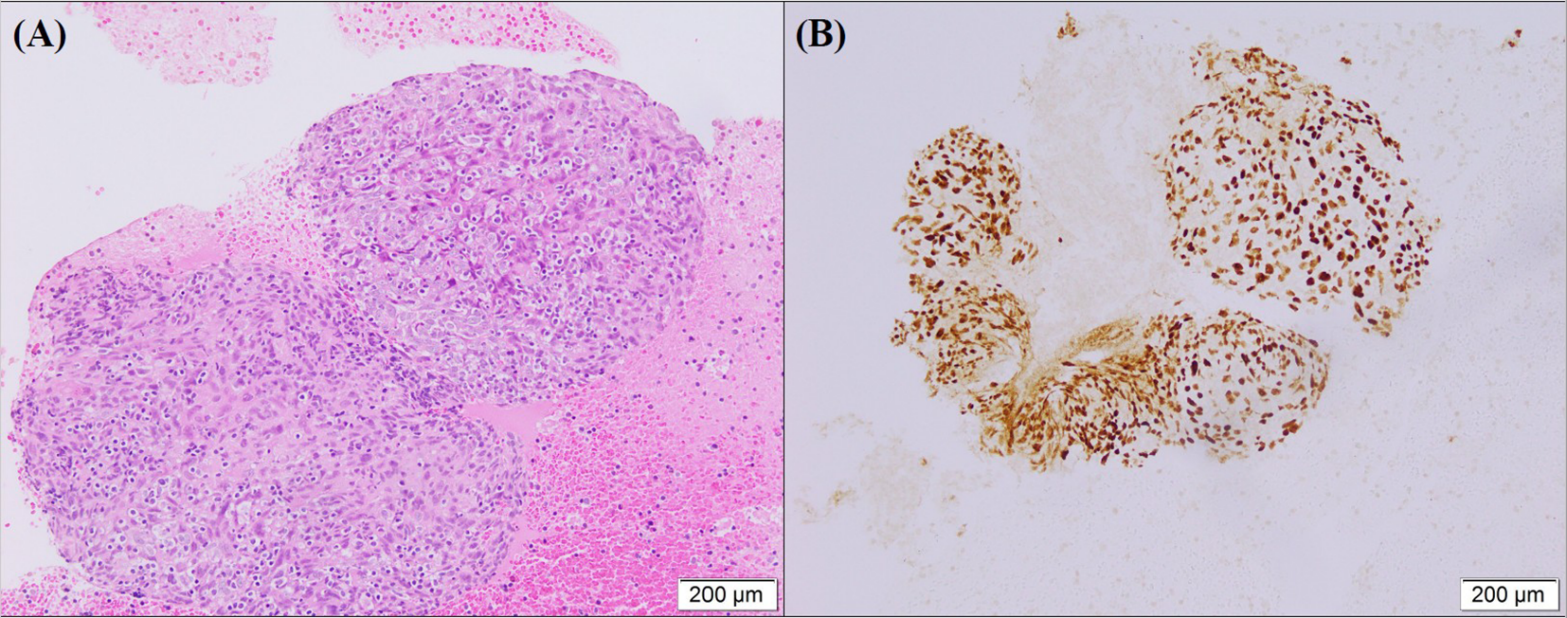
**(A)** HE staining show tumor cells arranged in a nest-like structure. The nuclei of the cancer cells were round or oval with prominent nucleoli, and lymphocyte infiltration was observed in the background. **(B)** EBER is positive *in situ* hybridization in tumor cells.

A 57-year-old southern China female was admitted to our hospital in January 2019 with a cough. The patient denied significant medical, surgical, or family history of malignancy, and had no smoking history. Chest computed tomography (CT) scan revealed a mass in the right hilum of the lung measuring approximately 4.6 x 3.7 cm, and a nodule in the medial segment of the right middle lobe measuring approximately 1.8 x 1.6 cm (**Figure 1A**). Multiple mediastinal lymph nodes of varying sizes were also observed. Serological examinations revealed an elevated EBV-DNA level of 1.01 × 10^4^ copies/mL, and cardiac, hepatic, and renal function tests, as well as tumor markers, were within normal limits. Tumor tissue obtained via fiberoptic bronchoscopy biopsy exhibited nest-like structures (**Figure 2**). The cancer cells displayed round to oval nuclei with distinct nucleoli, accompanied by lymphocyte infiltration. These histological features confirmed the diagnosis of pLELC, supported by positive Epstein-Barr virus encoded small RNAs(EBER) *in situ* hybridization. PD-L1 expression was assessed during the initial histopathological workup, revealing strong positivity with a Tumor Proportion Score (TPS) of 80%. Enhanced abdominal CT and brain magnetic resonance imaging(MRI) scans were normal, with no evidence of nasopharyngeal mucosal thickening, ruling out metastatic lung cancer from nasopharyngeal carcinoma. Based on the laboratory findings, chest scan and pathological results, the diagnosis was confirmed as pLELC of the right lung, staged as cT4N2M0, IIIB (Eastern Cooperative Oncology Group Performance Status score of 1) according to the 9th edition of the AJCC/UICC staging system.

The patient was diagnosed with unresectable, locally advanced, centrally located lung cancer. Following evaluation by thoracic surgery, surgical intervention was deemed unsuitable. After confirming eligibility criteria (no prior treatment and absence of chemotherapy contraindications), the patient received treatment with the GP regimen (gemcitabine 1250 mg/m² on days 1 and 8, plus nedaplatin 75 mg/m² on day 1) every three weeks, commencing on February 3, 2019. After four cycles induction chemotherapy of GP regimen, the follow-up chest CT scan indicated that the tumor had decreased in size (**Figure 1C**). The treatment response was assessed as PR according to the Response Evaluation Criteria in Solid Tumors (RECIST). Subsequent multidisciplinary team discussion concluded that thoracic surgery deemed the tumor unresectable due to its location close to the pulmonary hilum with vascular involvement, which presented significant technical challenges and carried a high risk of residual disease. Consequently, sequential radical radiotherapy was recommended as the definitive local treatment. Subsequently, the patient underwent radiotherapy targeting the primary chest tumor and the regional lymph nodes, with dose of 63 Gy/34 fractions. There were no grade 3-4 adverse reactions during the treatment of the patients (**Figure 1D**). Complete response (CR) was confirmed by post-radiotherapy surveillance CT​(**Figure 1E**). The patient was subsequently subjected to regular long-term follow-up, and as the most recent chest CT scan performed in July 2024, no signs of tumor recurrence were observed (**Figure 1F**).

## Discussion and conclusions

pLELC is commonly found in younger, non-smoking individuals from Southeast Asia. Patients with pLELC often show positive blood EBV-DNA tests, and pathological evaluation should include *in situ* hybridization for EBER. To our knowledge, this is the first case report of induction chemotherapy and sequential radiotherapy for locally advanced Stage IIIB pLELC. In this case, the patient with unresectable locally advanced pLELC achieved complete tumor suppression and clinical CR following GP regimen and sequential radiotherapy, providing an effective and safe treatment option for patients with locally advanced unresectable pLELC.

There is no consensus on the treatment principles of pLELC due to its low morbidity and lack of clinical trials. Accordingly, treatment strategies for pLELC are primarily based on the therapeutic principles for lung squamous cell carcinoma (1). For patients with locally advanced squamous cell lung cancer, radical concurrent chemoradiotherapy is the preferred treatment option (7). However, pLELC is not entirely equivalent to lung squamous cell carcinoma. Due to lack of study, particularly prospective studies, the effectiveness of radical concurrent chemoradiotherapy for pLELC has yet to be confirmed. pLELC shares certain pathological similarities with NPC and is closely linked to EBV infection. Both pLELC and NPC demonstrate significant lymphocytic infiltration within their tumor microenvironments (1, 8). The standard treatment for locally advanced NPC is induction chemotherapy followed by radical concurrent chemoradiotherapy. This approach aims to reduce tumor size and potentially downstage the disease before radical radiotherapy. A study has shown that for locally advanced NPC, induction chemotherapy followed by radiation alone provides a progression-free survival (PFS) rate comparable to concurrent chemoradiotherapy over three years (6). Although there are no available cases or studies comparing induction chemotherapy followed by radiotherapy in pLELC, it suggests that induction chemotherapy followed by radiotherapy may demonstrate non-inferiority to concurrent chemoradiotherapy in the treatment of pLELC. Therefore, in this case, a treatment approach based on strategies for locally advanced NPC was also adopted. Induction chemotherapy was first administered to achieve PR, followed by radical radiotherapy aimed at CR. In clinical practice, some patients may be reluctant to undergo first-line radiotherapy due to adverse reactions related to radiotherapy, such as radiation pneumonia (9, 10). For patients hesitant about radiotherapy, induction chemotherapy followed by radiotherapy provides a viable alternative to concurrent chemoradiotherapy. While its efficacy in locally advanced pLELC remains unstudied, this case demonstrated favorable outcomes with a treatment approach adapted from locally advanced NPC, involving induction chemotherapy followed by radical radiotherapy. This suggests potential clinical benefits of this regimen for locally advanced pLELC.

Previous studies have indicated that the GP regimen is an effective first-line chemotherapy option for advanced pLELC patients (11), demonstrating encouraging results in treating this cancer subtype. However, concurrent chemoradiotherapy utilizing the GP regimen is generally contraindicated due to an elevated risk of radiation pneumonitis (12). Studies have shown that concurrent radiotherapy with the TP regimen offers better safety compared to the GP regimen, suggesting it may be a viable alternative treatment option (11). However, the efficacy of the TP regimen is not as well-established as that of the GP regimen. Therefore, in this case, the patient received neoadjuvant chemotherapy with the GP regimen followed by radical radiotherapy. The follow-up chest CT showed a reduction in tumor size after four cycles of the GP regimen, confirming the efficacy of GP regimen for locally advanced pLELC and reducing the adverse effects associated with concurrent chemoradiotherapy. Although this case suggests that the GP regimen may be effective as induction therapy, no clinical trials have directly compared its advantages and disadvantages to other regimens for neoadjuvant chemotherapy. It remains unclear whether the GP regimen is more effective or safer than other chemotherapy regimens when used in induction chemotherapy, or whether it holds any particular advantage over radical concurrent chemoradiotherapy. Additionally, neoadjuvant therapy for NSCLC typically involved 3–4 cycles, while NPC generally involved 2 cycles (13, 14). In this case, the patient achieved a PR after the four cycles of neoadjuvant chemotherapy, and subsequent radical radiotherapy resulted in a CR of the lesion. The optimal number of cycles for induction chemotherapy remained unclear.

In the context of immunotherapy, pLELC demonstrates significantly higher PD-L1 expression compared to common NSCLC subtypes, largely attributable to EBV-induced immunomodulation—as exemplified by the 80% TPS observed in this case (15). A recent retrospective analysis revealed significantly prolonged median progression-free survival (mPFS) in advanced pLELC patients treated with chemoimmunotherapy versus chemotherapy alone (11.8 vs. 6.9 months, P=0.007) (16). Furthermore, neoadjuvant/adjuvant chemoimmunotherapy demonstrates enhanced efficacy in resectable pLELC, including higher rates of major pathological response (MPR) and pathological complete response (pCR) (17).These findings collectively suggest that combining immunotherapy with chemotherapy may improve survival outcomes across all stages of pLELC. Nevertheless, definitive validation remains limited by the rarity of this entity, underscoring the need for large-scale prospective trials. Additionally, there remained approximately a 20-30% chance of recurrence after completing treatment for NPC (6, 18). Whether pLELC also had a higher risk of recurrence and whether maintenance therapy was necessary remained unresolved issues. In summary, further research was needed to determine the most effective regimen for neoadjuvant chemotherapy. However, due to the rarity of this tumor, conducting such research or clinical trials faced significant challenges.

Radiotherapy is a critical component in the treatment of EBV-associated tumors, particularly NPC, where it serves as the primary therapeutic approach. The effectiveness of radiotherapy in NPC is attributed not only to the anatomical characteristics of the nasopharynx but also to unique pathological features of the tumor microenvironment, including extensive lymphocyte infiltration (2, 3, 19). EBV-associated gastric cancer in a mouse xenograft modelalso shows a favorable response to radiotherapy (20). As previously mentioned, both pLELC and NPC are presented as poorly differentiated squamous cells with rapid proliferation, high metastatic potential, and significant invasiveness. These characteristics may contribute to pLELC’s heightened sensitivity to radiation. Additionally, studies have shown that pLELC shares significant genetic similarities and the EBV genome with NPC (21), suggesting that, like NPC, pLELC may also exhibit high radiosensitivity. A retrospective study on locally advanced pLELC showed that both the chemoradiotherapy group and the radical surgery plus adjuvant chemoradiotherapy group had better clinical outcomes compared to the radical surgery plus adjuvant chemotherapy group (22). This implies that radiotherapy could provide favorable therapeutic outcomes for patients with unresectable locally advanced pLELC. The potential value of radiotherapy in pLELC remains inadequately investigated and underutilized in both research and clinical practice. Regarding dosage, the National Comprehensive Cancer Network guidelines recommend 60–70 Gy in 30 fractions for locally advanced NSCLC and 70–70.2 Gy in 35 fractions for locally advanced NPC. Since pLELC is theoretically more radiosensitive than NSCLC, further research is needed to identify the optimal balance between efficacy and safety in this setting. Determining the optimal radiation dosage and ideal number of cycles for radical radiotherapy following neoadjuvant chemotherapy or radical concurrent chemoradiotherapy in patients with locally advanced pLELC requires additional research to fully maximize the benefits of radiotherapy.

EBV exhibits a unique duality in pLELC. On one hand, pLELC demonstrates a favorable prognosis compared with other NSCLC subtypes lacking EBV infection—an effect that has been hypothesized to stem from the robust lymphocytic infiltration driven by chronic EBV infection, which may indirectly enhance chemosensitivity (4, 23). On the other hand, EBV viral proteins such as LMP1 interact with host cellular machinery to drive cell cycle progression, promote anti-apoptotic signaling, and facilitate immune evasion, thereby potentially accelerating tumor progression and metastasis (24, 25). Therapeutically, this complexity is particularly salient: GP regimen, centered on gemcitabine, has demonstrated clear clinical benefit in EBV-associated malignancies including pLELC through multiple interrelated mechanisms. Gemcitabine has been shown to activate the ATM/Chk2 DNA damage response pathway and upregulate EBV lytic-phase initiators, thereby inducing viral lytic reactivation and direct tumor cell lysis (26, 27); concurrently, gemcitabine-mediated DNA damage sensitizes tumor cells to radiotherapy. Intriguingly, the ostensibly oncogenic LMP1 may under certain conditions enhance tumor cell susceptibility to radiation-induced apoptosis, further increasing radiotherapy sensitivity (28).

This report is limited by its single-case design, which restricts the generalizability of our findings and precludes definitive conclusions regarding the efficacy of GP induction chemotherapy combined with definitive radiotherapy in pLELC. To strengthen validation of this regimen, future studies should include larger cohorts of pLELC patients, ideally through prospective multicenter trials or registry analyses. Moreover, current insights into the molecular mechanisms are largely extrapolated from other EBV-associated malignancies and from the general effects of chemotherapeutic agents; no studies to date have specifically investigated the molecular pathways underlying the GP regimen in pLELC. Subsequent work should therefore focus on elucidating the specific interactions between GP agents and EBV—such as gemcitabine-induced EBV lytic reactivation, ATM/Chk2 pathway activation, and cisplatin-mediated antigen release—to clarify the molecular basis of durable remission. Addressing these limitations is essential to confirm both the reproducibility and mechanistic rationale of this novel multimodal treatment strategy. Moreover, this case did not include serial monitoring of plasma EBV DNA, precluding any analysis of its temporal dynamics. This omission reflects the current lack of consensus on the clinical utility of EBV DNA kinetics in pLELC management, as well as cost-benefit considerations at the time of treatment. Emerging data—study demonstrating that dynamic changes in EBV DNA levels can serve as both prognostic biomarkers and predictors of therapeutic response—underscore the potential value of incorporating routine EBV DNA surveillance into future trials (29). Lastly, it is important to note that immune checkpoint inhibitors had not yet become standard of care for rare EBV-associated malignancies like pLELC in 2019, which limited our ability to explore combined immunochemoradiotherapeutic strategies in this patient.

In conclusion, this case demonstrated that sequential chemoradiotherapy regimen brings significant and durable survival benefit to patients with unresectable locally advanced pLELC. Simultaneously, extensive research is still requisite to delve into and establish a consensus regarding the standard therapeutic approach for unresectable locally advanced pLELC.

## Data Availability

The original contributions presented in the study are included in the article, further inquiries can be directed to the corresponding author/s.
